# Effectiveness of Botulinum Neurotoxin in Treatment of Scoliosis among Children and Adolescents: A Systematic Review and Meta-Analysis

**DOI:** 10.3390/children9101505

**Published:** 2022-10-01

**Authors:** Yu-Chi Su, Yu-Ching Lin, Pei-Chun Hsieh, Chung-Lun Liao, Yao-Hong Guo

**Affiliations:** 1 Department of Physical Medicine and Rehabilitation, National Cheng Kung University Hospital, College of Medicine, National Cheng Kung University, Tainan City 70428, Taiwan; 2 Department of Physical Medicine and Rehabilitation, College of Medicine, National Cheng Kung University, Tainan City 70101, Taiwan; 3 School of Medicine, College of Medicine, National Cheng Kung University, Tainan City 70101, Taiwan

**Keywords:** botulinum neurotoxins, scoliosis, meta-analysis, cerebral palsy

## Abstract

Scoliosis refers to a three-dimensional deviation in the axis of the spine. Muscle imbalance is believed to play a role in scoliosis. Botulinum neurotoxin (BoNT) can reduce muscle overactivity and may have the potential to ameliorate spinal scoliosis. This study investigated the effectiveness of intramuscular BoNT injection in vertebral curve correction and reviewed the possible influencing factors. PubMed, Medline, Cochrane Central Register of Controlled Trials, Web of Science, Airiti Library, and Index of the Taiwan Periodical Literature System databases were searched from inception until 7 September 2022 for eligible studies. The main outcome was the change in Cobb angle after BoNT application. Subgroup analysis was conducted according to differences in study designs, etiology of scoliosis, and methods used for target muscle selection. We enrolled three studies including 31 participants aged between 2 and 18 years. The meta-analysis revealed no significant reduction in the Cobb angle after BoNT injection (standardized mean difference, −0.783, 95% CI, −2.142 to 0.576). Study designs (*p* = 0.011) and methods used for target muscle selection (*p* = 0.017) but not etiology of scoliosis (*p* = 0.997) reached statistical significance between subgroups. In conclusion, the current meta-analysis does not support the application of BoNT in children and adolescents with scoliosis. However, a decisive conclusion could not be made due to high between-study heterogeneity and small sample size. More randomized controlled trials with appropriate target muscle selection and standard outcome measurement should be conducted to examine the efficacy of botulinum neurotoxin in treating scoliosis. INPLASY ID: INPLASY202290031.

## 1. Introduction

According to the Scoliosis Research Society, patients with a curvature of the spine in the frontal plane (Cobb angle) over 10° and axial rotation are clinically diagnosed as having scoliosis [1]. Scoliosis is categorized into 2 major types: idiopathic and nonidiopathic. The nonidiopathic type includes congenital, neuromuscular, and mesenchymal scoliosis. If nonidiopathic disorders are excluded, idiopathic scoliosis may be diagnosed [2]. The etiology of idiopathic scoliosis remains inconclusive, and current studies have proposed several hypotheses such as abnormalities in the paraspinal musculature, growth hormone secretion, melatonin secretion, estrogen receptor structure and function, connective tissue structure, vestibular function, and tissue calmodulin [1,3,4,5,6].

Conservative approaches are usually applied before surgery, and the options and goals of conservative treatment depend on the underlying etiology of scoliosis [7]. If conservative therapies fail, surgery may be considered. However, considerable surgical complication rates were still reported regardless of the etiology of scoliosis [8,9]. Moreover, spinal fusion surgery is not favored before the age of 8 to 10 years because their the lungs and spine are not developed fully [10]. Hence, an alternative or complementary treatment to prevent or postpone surgery should be invented.

Botulinum neurotoxin (BoNT) can induce the paralysis of striated muscles by blocking the release of acetylcholine and inhibiting the transmission of alpha motor neurons [11]. BoNT has been proven to be effective and safe in treating overactive muscles including dystonia and spasticity [11,12,13]. In some neuromuscular disorders such as cerebral palsy, the muscle imbalance around the spinal axis caused by spastic or flaccid muscles is believed to be associated with spinal deformity [14]. Previous studies have reported the presence of electrophysiological and radiological muscle imbalance in idiopathic scoliosis [15,16,17,18,19,20]. By modulating the hypertonic muscles in neuromuscular scoliosis and rebuilding the balance between muscle pairs in idiopathic scoliosis with BoNT, it is reasonable to expect the effect in reducing Cobb angles. Although several studies have evaluated the effectiveness of BoNT in scoliosis treatment, which is not labeled for such use currently, they have reported controversial results [21,22,23,24,25]. To date, no study has systemically evaluated the effectiveness of BoNT injection in scoliosis treatment.

In this study, we investigated the effectiveness of BoNT in scoliosis treatment among children and adolescents by conducting a systematic review and meta-analysis of published articles. In addition, we examined the moderators of effectiveness including the study design, etiology of scoliosis, and method used for target muscle selection. Because the muscle characteristics were different between neuromuscular and idiopathic scoliosis, we hypothesized that the etiology might be an important variable to the treatment effect. Moreover, the muscles responsible for the scoliosis might be discrepant among individuals. An accurate functional evaluation, such as electrical stimulation, to identify the target muscles might play a vital role in BoNT injection. We included both randomized controlled trials and nonrandomized controlled studies due to the small number of studies on this topic thus far.

## 2. Materials and Methods

This systematic review was conducted following the Preferred Reporting Items for Systematic Review and Meta-Analysis (PRISMA) guidelines (See File S1. for the PRISMA checklist) [26]. We registered the protocol on the International Platform of Registered Systematic Review and Meta-analysis Protocols (registration number: INPLASY202290031).

### 2.1. Eligibility Criteria

The inclusion criteria were as follows: (1) case arm including patients under 18 years old with scoliosis treated with BoNT; (2) control arm with patients under 18 years old not receiving BoNT or no control arm; and (3) randomized controlled trials or nonrandomized controlled studies.

We excluded conference proceedings as well as case reports due to the extremely high possibility of publication bias. In addition, studies that did not report the etiology of scoliosis were excluded.

### 2.2. Literature Search

We electronically searched PubMed, Medline, Cochrane Central Register of Controlled Trials, Airiti Library, Web of Science, and Index of the Taiwan Periodical Literature System databases for eligible studies by using a combination of “scoliosis” AND “botulinum toxin” as keywords. We identified studies that were published from inception to the present time, and the final search was conducted on 7 September 2022 (See File S1. For the full search strategy).

### 2.3. Study Selection and Data Extraction

Two authors independently reviewed the titles and abstracts of eligible studies after excluding duplicate articles. A data collection sheet was used to extract the following data from included studies: participants’ demographics, injection parameters, adverse events, Cobb angle, and other clinical or radiological outcomes. We contacted corresponding authors as necessary by email to retrieve missing data.

### 2.4. Quality Assessment

For randomized controlled trials, the risk of bias was examined using the Cochrane risk of bias tool [27]. We adopted the Joanna Briggs Institute Critical Appraisal Checklist to evaluate the nonrandomized controlled studies [28]. The results were shown in a risk of bias graph using Review Manager version 5.3 (Cochrane, London, UK).

### 2.5. Statistical Analysis

The primary outcome was the improvement in the Cobb angle after BoNT injection and represented by standardized mean differences (SMDs) and 95% confidence intervals (Cis). The Cobb angle before and after BoNT treatment were used to analyze the summary effect size. If the Cobb angle of the lumbar and thoracic curve were separately mentioned, the data were added together for meta-analysis, as described in a previous study [22]. The random-effects model was used for pooling of effect sizes. Subgroup analysis was conducted according to differences in study designs, etiology of scoliosis, and methods used for target muscle selection. A significant difference between effect sizes was defined by *p* < 0.05. I^2^ was used to grade the between-study heterogeneity. Cutoff values of 50% and 75% were used for low, moderate, and high heterogeneity [29]. Publication bias was examined by funnel plots and Egger’s test, and significance was reached when the two-tailed *p* value was <0.1 [30]. Comprehensive Meta-analysis Software version 3 was used for the analysis (Biostat, Englewood, NJ, USA).

### 2.6. Certainty of Evidence

For the primary outcome, we used the Grading of Recommendations Assessment, Development and Evaluation (GRADE) methodology to assess the certainty of Evidence. Because our study included articles other than randomized controlled trials, the results begin as moderate certainty. The final rating depends on publication bias, inconsistency, indirectness, overall risk of bias, and imprecision.

## 3. Results

### 3.1. Study Selection and Description

**Figure 1:**
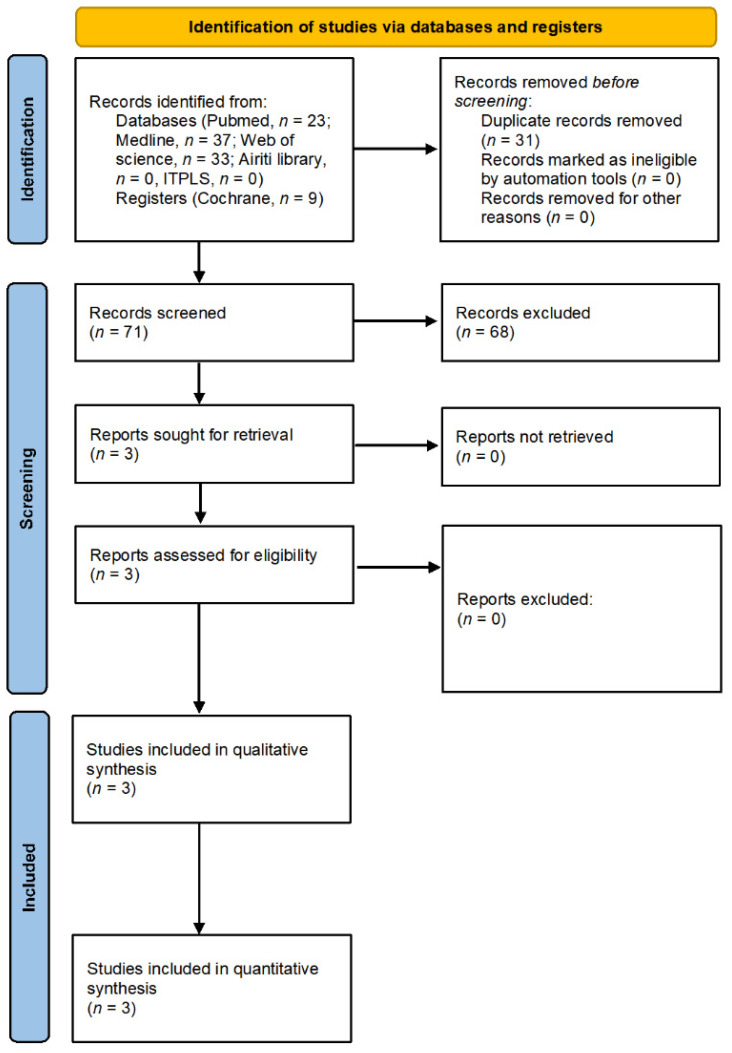
Literature screening process and results.

After initial search and duplicate removal, 71 studies were retrieved for screening. Three studies met our inclusion criteria, as indicated in the PRISMA flowchart (Figure 1). The main characteristics of the three studies included in this systematic review are listed in Table 1.

Two studies included patients with neuromuscular scoliosis. Nuzzo et al. evaluated a prospective case series of patients with a combination of neuromuscular disorders and scoliosis [21]. OnabotulinumtoxinA was administered to selected muscles that were believed to deteriorate the spine curvature. Wong et al. conducted a triple-blinded, randomized controlled trial including crossover treatment [22]. All the participants had a history of cerebral palsy, and the Gross Motor Function Classification System (GMFCS) score of the enrolled patients ranged from III to V. The exact formulation (or brand name) of BoNT used in the study was not reported; however, they might have used onabotulinumtoxinA because a similar protocol was reported in another study conducted by the same research team [22,23].

The last study focused on idiopathic scoliosis. Wong et al. examined a prospective case series of nine participants with adolescent idiopathic scoliosis (AIS) [23]. OnabotulinumtoxinA were administered on the concave side of the psoas major muscle under the guidance of ultrasonography and needle electric stimulation (Table 2).

In terms of adverse effects, Wong et al. reported that two patients with AIS experienced transient soreness after BoNT injection [23]. In another study conducted by Wong et al., one patient with cerebral palsy-related scoliosis died due to pneumonia [22]. This patient received 480 units of BoNT injection and two consecutive surgical interventions with a short interval between them.

### 3.2. Risk of Bias Assessment

**Figure 2:**
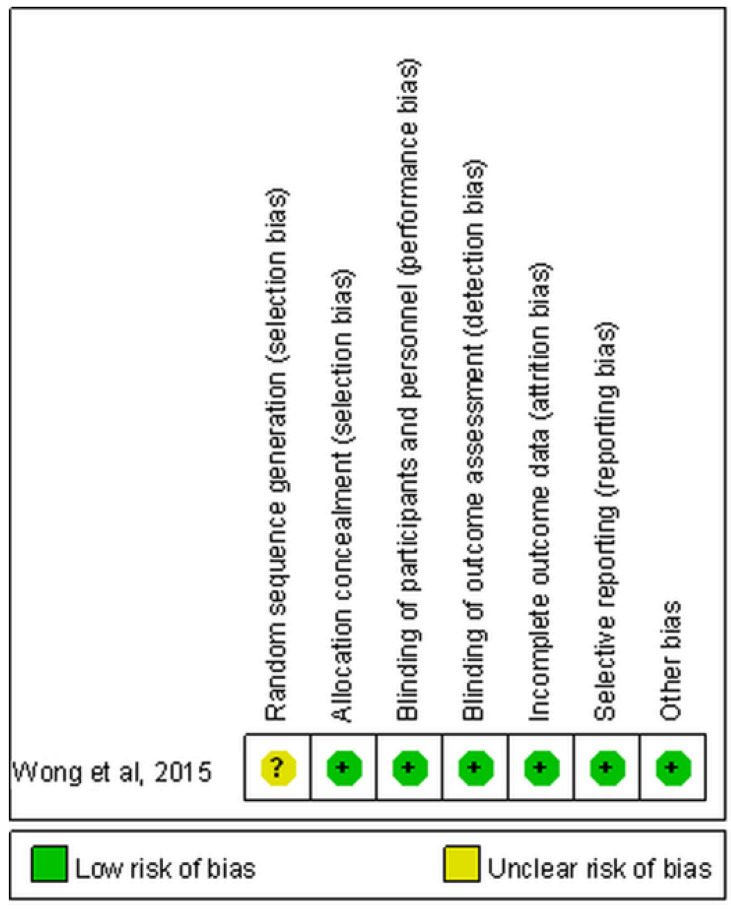
Summary for risk of bias of randomized controlled trial.

**Figure 3:**
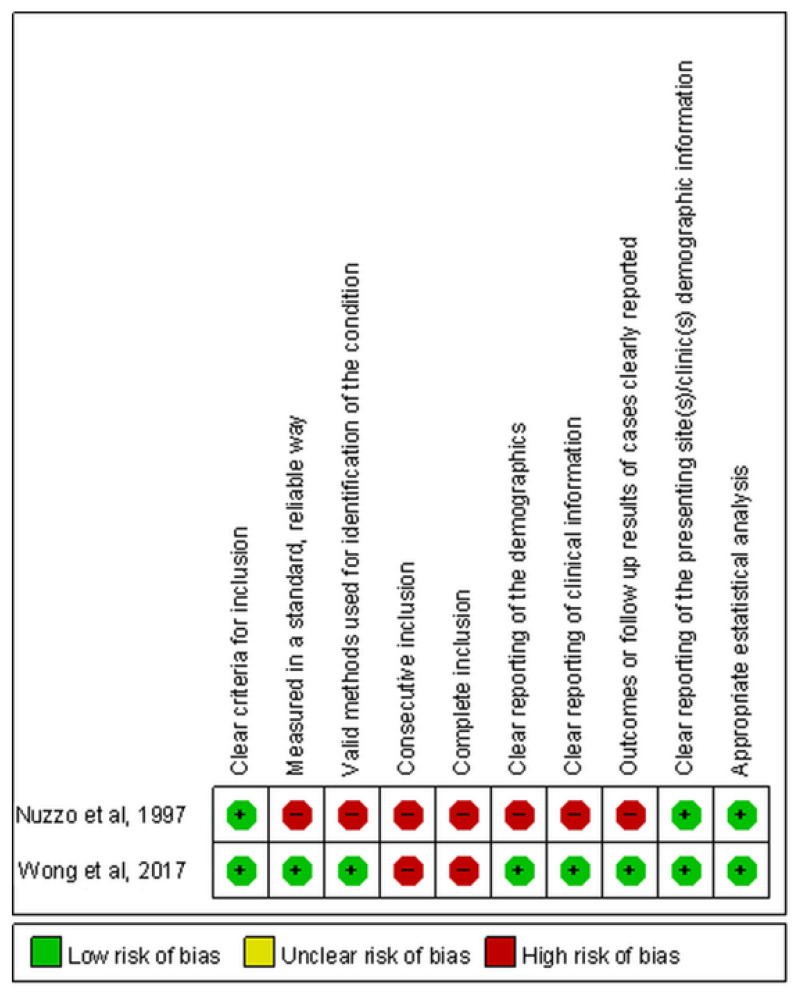
Summary for risk of bias of nonrandomized controlled studies.

The randomized controlled trial performed by Wong et al. had an unclear risk of selection bias because they did not mention details regarding the generation of the random sequence (Figure 2) [22]. In the other two studies, Nuzzo et al. did not identify the conditions of patients in a standard manner and did not clearly report demographics and clinical information [21]. Both Nuzzo et al. and Wong et al. did not indicate whether the cases were consecutively included or not (Figure 3) [21,23].

### 3.3. Results of Quantitative Synthesis

**Figure 4:**
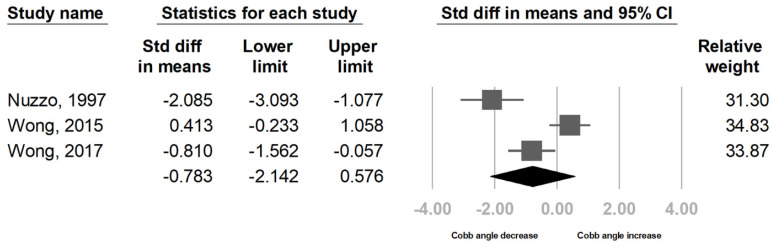
Forest plot of standardized mean differences in reduction in Cobb angle after treatment. Effect of individual studies were presented by squares. 95% CI was presented by lines. The summarized effect size was presented by diamond.

We performed a meta-analysis of three studies to examine the improvement in the Cobb angle 6 weeks after BoNT injection [21,22,23]. One of them was a randomized controlled trial [22], and the other two were case series [21,23]. The meta-analysis comprised 31 patients. The results of the meta-analysis revealed that BoNT injection did not significantly reduce the Cobb angle 6 weeks after intervention (SMD, −0.783, 95% CI, −2.142 to 0.576, I^2^ = 89%; Figure 4) with high between-study heterogeneity.

**Figure 5:**
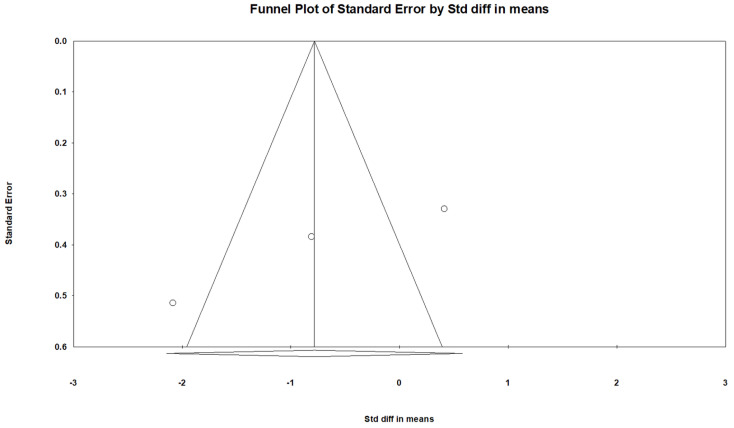
Funnel plot of the included articles. The summarized effect size was showed by the diamond, and each dot indicates a single study.

For subgroup analysis, grouping by etiology of scoliosis resulted in two subgroups. The idiopathic scoliosis group comprised one study [23], and the neuromuscular group included two studies [21,22]. Subgroup analysis revealed a significant decrease in Cobb angle in idiopathic scoliosis but not neuromuscular scoliosis (Table 3). However, the difference between subgroups did not reach statistical significance (*p* = 0.997). For methods used for target muscle selection, the electrical stimulation group included one study [21], and the beforehand group comprised two articles [22,23]. The electrical stimulation group but not beforehand group showed a significant decrease in Cobb angle after intervention, and the difference between the two subgroups was significant (*p* = 0.017, Table 3). For different study designs, one trial was a randomized controlled trial [22], and two were nonrandomized controlled studies [21,23]. Nonrandomized controlled studies but not randomized controlled trials showed a significant decrease in Cobb angle after injection, and the difference between subgroups were significant (*p* = 0.011, Table 3). No remarkable publication bias was detected according to the funnel plot and Egger’s test (*p* = 0.155, Figure 5).

### 3.4. Certainty of Evidence

The certainty of evidence of the improvement of Cobb angle after treatment revealed very low quality of evidence. High risk of bias, large CI, and significant between-study heterogeneity resulted in the downgrade in level. The details were presented in Table 4.

## 4. Discussion

The results of this meta-analysis revealed that intramuscular BoNT injection did not reduce the Cobb angle of scoliosis at the 6-week follow-up. However, significant heterogeneity between studies were noted, which might result from different study designs and different methods for muscle selection. Nonrandomized controlled studies showed a significantly larger treatment effect than randomized controlled trials. Moreover, selecting the target muscle for injection by electrical stimulation revealed a significantly larger decrease in Cobb angle than injecting BoNT into fixed muscles decided beforehand.

According to our findings, the success or failure of scoliosis treatment with BoNT might depend on the accuracy of target muscle selection. The effect of BoNT should be better in hypertonic muscles rather than fixed contracture due to its mechanism of action [31]. Hence, distinguishing muscle overactivity and fixed shortening by tools such as electromyography may also increase the treatment effect [32]. Likewise, the muscle stimulation used by Nuzzo et al. might play a role in preventing the administration of BoNT to the part of muscles with fixed contracture. In summary, we hypothesize that the introduction of functional evaluation of muscles, such as through electrical stimulation or dynamic electromyography, for guiding BoNT injection may help identify the pathological component of concave-side paraspinal muscles and optimize the treatment efficacy of BoNT in scoliosis. Randomized controlled trials with larger number of participants are warranted to prove our findings.

Different etiology of scoliosis did not cause a significantly different treatment effect in our meta-analysis. However, this may be a false negative result due to insufficient data. The type of scoliosis may still have an impact on the effect of BoNT. Patients with cerebral palsy scoliosis, a major subgroup of neuromuscular scoliosis, can present with both asymmetric muscle tone and weakness in paraspinal and intercostal muscles [33]. During the period of growth, in contrast to the fast-growing skeleton, the relative shorter muscle might increase spasticity. This phenomenon might further aggravate scoliosis in cerebral palsy [34]. BoNT may thus carry out its treatment effect by relaxing the spastic muscles. Unlike neuromuscular scoliosis, the pathomechanic role of paravertebral muscles in AIS is still under debate [35]. Here, we hypothesize the pathological role of muscle imbalance in AIS by summarizing up-to-date research. Paraspinal muscles at the convex side of the curved spine have been found to be stronger electrophysiologically, radiologically, and histologically. Electrophysiologically, a significantly higher amplitude of motor unit potentials was observed on the convex side of the scoliotic curve [16,18]. Radiologically, a study surveying idiopathic thoracic scoliosis through magnetic resonance imaging revealed an enlarged muscle volume of the thoracic multifidus, semispinalis, and rotator muscles on the convex side relative to the concave side at the apex, upper-end, and lower-end vertebra level [19]. Histologically, a study observed that the fatty infiltration rate was considerably higher in the concave-side muscles, and the authors concluded that these abnormalities may be explained by paraspinal muscle adaptation to the increased loading demand on the convex side of the curve [18]. By contrast, one study reported that the cross-sectional area of the psoas major muscle was larger on the concave side of the lumbar scoliosis before skeletal maturity, and higher psoas major muscle imbalance at the apical vertebral level was correlated to a larger Cobb angle [20]. Considering that the muscle volume and cross-sectional area are proportional to muscle strength [36], abnormally stronger psoas major muscles on the concave side might play a pathomechanic role in AIS. The stronger erector spinae and transversospinalis muscles on the convex side might be a compensatory response to chronic high load demand. BoNT may decrease the Cobb angle by decreasing the abnormal strength of the psoas muscle on the concave side of the spine, and this hypothesis is supported by the results reported by Wang et al. [23]. Whether other muscles, such as the quadratus lumborum, play a similar role as the psoas major should be examined in future studies [35]. To sum up, future studies are necessary to clarify the relationship between etiology of scoliosis and the treatment effect of BoNT.

Despite the antispastic effect, BoNT could also modify the pain sensation and muscles properties such as muscles tone and stiffness [37]. Moreover, muscle atrophy with necrosis and fibrosis of muscle fibers after BoNT injection had been observed in both human and animal models [38]. These changes may interfere with the exercise performance of the scoliosis patient. However, scoliosis-specific exercise plays an important role in AIS and may have the potential to reduce Cobb angle and improve trunk balance and quality of life [39]. The 2016 Scientific Society on Scoliosis Orthopaedic and Rehabilitation Treatment (SOSORT) guideline also recommended patients with AIS to remain active in sports activities. Patients performing sporting activities can better identify their scoliotic curve, get better psychological outcomes, and reveal higher self-esteem than the sedentary group [40]. The interaction between BoNT and exercise in scoliosis also needs future studies to clarify.

The safety of applying BoNT in scoliosis patients is of the greatest concern. One study enrolled in this systematic review reported a case of possible serious adverse event of pneumonia that resulted in death [22]. The authors concluded that a relationship between BoNT and death cannot be ruled out. The safety of applying BoNT in higher GMCSF score patients are still under debate. In clinical treatment of pediatric spasticity, the recommended dose of onabotulinumtoxinA by the manufacturer was 3 to 6 units/kg body weight in the upper limbs and 4 to 6 units/kg body weight in the low limbs [41]. Some studies have reported that systemic adverse events such as lower respiratory tract illnesses were positively correlated to GMFCS level and dose of BoNT, and one study even proposed that cerebral palsy patients of GMFCS level V should not be treated with BoNT [42]. By contrast, other studies for patients of GMFCS level V reported no adverse effects with onabotulinumtoxinA doses of 15 to 20 units/kg body weight and less than a total dose of 300 units [43,44]. Summarizing current studies, onabotulinumtoxinA dose of 3 to 10 units/kg body weight with a total dose of less than 300 units might be safe, applicable, and promising in treating scoliosis. However, this hypothesis needs further experimental verification.

This study has several strengths. First, we were the first to conduct a meta-analysis focusing on the application of BoNT in scoliosis. Second, we were the first to reveal the moderator effect of methods used for target muscle selection. Third, we were the first to reveal a significantly larger treatment effect of nonrandomized controlled studies than randomized controlled trials. This finding highlights the potential bias of nonrandomized controlled studies as well as encourages future researchers to carry out rigorous trials to clarify the effectiveness of BoNT in scoliosis.

This study has some limitations. First, because of the scarcity of published articles and small sample size, we could not exclude the possibility of type II errors. Second, because of the high heterogeneity of the studies in this field, such as study designs, etiology and type of scoliosis, dosage of BoNT, and muscle selection, the study results should be interpreted cautiously. Third, all enrolled studies reported the rate of bracing treatment of participants [21,22,23], but none of them described the dosage, quality of bracing, and patient compliance, which are positively correlated with bracing efficacy [1]. Elucidating the possible relationship between the efficacy of bracing and BoNT is crucial. Fourth, we included both randomized controlled trials and nonrandomized controlled studies, which impeded the results of our meta-analysis. Fifth, we could not assess the effect of BoNT in preventing or postponing surgery due to insufficient data. This knowledge gap remained unanswered so far, and future studies are warranted to fill this gap. Finally, cerebral palsy-related scoliosis was believed to be a secondary musculoskeletal deformity that resulted from several negative etiologies, such as upper motor neuron disease, impaired motor control, muscular imbalance, and functional restriction [22]. In addition, the scoliogenic muscles of AIS were not well understood [23,35]. However, we could not investigate the moderator effects of these factors on the efficacy of BoNT in scoliosis not only due to the small number of studies currently, but also due to the complex nature of scoliosis. Considering the aforementioned limitations and the limited choice of conservative treatments for scoliosis thus far, future studies should investigate the effects of BoNT in scoliosis.

## 5. Conclusions

Current evidence does not support the administration of BoNT in children and adolescents with scoliosis. However, definite conclusions cannot be drawn due to high between-study heterogeneity and a small sample size. Randomized controlled trials with a larger number of participants and more homogenous participants are warranted to verify the effectiveness and safety of BoNT in treating scoliosis.

## Figures and Tables

**Table 1:** The detailed characteristics of all included study.

**Research**	**Country**	**Study Design**	**Age at the Time Receiving BTI (Years)**	**Gender (Male/Female)**	**Etiology of Scoliosis**	**Level of Scoliosis (T/L/TL)**
Nuzzo et al., 1997 [21]	USA	Case series	9.08 (2–18)	4/8	Neuromuscular scoliosis	NR
Wong et al., 2015 [22]	Denmark	Randomized controlled trial	11.07 (3.9–17.8)	5/5	Neuromuscular scoliosis (CP related)	0/0/10
Wong et al., 2017 [23]	Denmark	Case series	12.39 (8.44–14.6)	1/8	Idiopathic scoliosis (AIS)	2/0/7
**Research**	**Scoliosis Type (C-Shaped/S-Shaped)**	**Risser Score (Score/Number of Patients)**	**GMFCS (Score/Number of Patients)**	**Post-BTI Follow-up**	**Bracing before BTI (Yes/No)**	**Other Baseline Characteristics**
Nuzzo et al., 1997 [21]	11/1	NR	NR	5.2 (2–15) months	12/0	Worsening scoliosis, unresponsive or intolerant to bracing, surgery indicated
Wong et al., 2015 [22]	7/3	NR	III/2, IV/3, V/5	6 weeks	10/0	History of CP, 2–18 y/o, brace treated
Wong et al., 2017 [23]	2/7	0/3, 1/1, 2/1, 3/0, 4/4	NR	6 weeks	7/2	History of AIS, Cobb angle above 10 degrees, 10–14 y/o, no modification in physiotherapy and bracing throughout the study

Results are presented as mean (range); AIS: adolescent idiopathic scoliosis; BTI: botulinum neurotoxin type A injection; CP: cerebral palsy; NR: not reported; T: thoracic; L: lumbar; TL: thoracolumbar; GMFCS: Gross Motor Function Classification System; y/o: years old.

**Table 2:** The summarized extracted data from the included studies.

**Study**	**Number of BTI and Duration of Continuous BTI (Month)**	**Commercial Forms**	**Injection Dose (U)**	**Dilution Method**
Nuzzo et al., 1997 [21]	0 patients received repeat BTI	OnabotulinumtoxinA	6 U/kg body weight per muscle, with upper limit of 100 U per muscle	NR
Wong et al., 2015 [22]	0 patients received repeat BTICrossover at 6 months after injection	NR	1 (100 U), 2 (50 U), and 3 (30 U) injection sites for respective muscles, with upper limited of 600 U	100 U/mL with normal saline
Wong et al., 2017 [23]	0 patients received repeat BTI	OnabotulinumtoxinA	3 injection sites for target muscles, total not exceeding 100 U per muscle	NR
**Study**	**Comparative Regimen**	**Injected Muscles**	**Injection Technique**	**Outcome**
Nuzzo et al., 1997 [21]	NR	Concave-side paraspinal muscles	Image and needle electrode muscle stimulation	Cobb angle
Wong et al., 2015 [22]	Normal saline	Concave-side Iliopsoas, quadratus lumborum, erecter spinae	Ultrasound	Cobb angle, NM classification, PedsQL, Subsequent surgery, Adverse events
Wong et al., 2017 [23]	NR	Concave-side psoas major	Ultrasound, needle electric stimulation	Cobb angle, NM classification, Rib vertebral angle, Subsequent surgery, Adverse events

BTI: botulinum neurotoxin type A injection; NR: not reported; U: unit; NM classification: Nash and Moe’s classification; PedsQL: Pediatric Quality of Life Inventory.

**Table 3:** Results of subgroup analysis.

		Standardized Mean Difference and 95% Confidence Intervals	*p* Value between Subgroups
Etiology	Neuromuscular scoliosis	−0.805 (−3.252, 1.642)	0.997
	Idiopathic scoliosis	−0.810 (−1.562, −0.057)	
	Total	−0.783 (−2.142, 0.576)	
Methods for muscle selection for injection	Decided beforehand	−0.183 (−1.380, 1.015)	0.017
	Decided by electrical stimulation	−2.085 (−3.093, −1.077)	
	Total	−0.783 (−2.142, 0.576)	
Study design	Nonrandomized controlled studies	−1.402 (−2.648, −0.155)	0.011
	Randomized controlled trial	0.413 (−0.233, 1.058)	
	Total	−0.783 (−2.142, 0.576)	

**Table 4:** Certainty of evidence for improvement of Cobb angle after treatment.

Quality Assessment						Summary of Findings, SMD (95% CI)		
Number of Participants (Studies)	Risk of Bias	Inconsistency	Indirectness	Imprecision	Publication Bias	Control	Botulinum Neurotoxin	Certainty of Evidence
31 (3)	Serious limitation ^a^	Serious limitation ^b^	No serious limitation ^c^	Serious limitation ^d^	Undetectable ^e^	- ^e^	−0.783 (−2.142, 0.576) ^f^	Very Low ⨁◯◯◯

CI: confidence interval; SMD: standardized mean difference; ^a^ One study scored high risk of bias in most aspects during assessment; ^b^ The I^2^ was over 50%; ^c^ No indirectness was detected in this outcome; ^d^ The upper and lower limit of 95% CI pointed in opposite directions; ^e^ There were insufficient data available so far to calculate the summary finding of this column; ^f^ This was calculated by pooling the botulinum neurotoxin group of the three studies, comparing Cobb angle before and after treatment.

## Data Availability

No new data were created in this study.

## Supplementary Materials

The following supporting information can be downloaded at: https://www.mdpi.com/article/10.3390/children9101505/s1, File S1: PRISMA checklist and search strategies for systematic review and meta-analysis.
